# MicroRNAs in Salivary Exosome as Potential Biomarkers of Aging

**DOI:** 10.3390/ijms160921294

**Published:** 2015-09-07

**Authors:** Tatsuya Machida, Takaaki Tomofuji, Daisuke Ekuni, Takayuki Maruyama, Toshiki Yoneda, Yuya Kawabata, Hirofumi Mizuno, Hisataka Miyai, Muneyoshi Kunitomo, Manabu Morita

**Affiliations:** 1 Department of Preventive Dentistry, Okayama University Graduate School of Medicine, Dentistry and Pharmaceutical Sciences, 2-5-1 Shikata-cho, Kita-ku, Okayama 700-8558, Japan; E-Mails: tomofu@md.okayama-u.ac.jp (T.T.); dekuni7@md.okayama-u.ac.jp (D.E.); de17057@s.okadai.jp (T.Y.); de18019@s.okayama-u.ac.jp (Y.K.); pc2o46pj@okayama-u.ac.jp (H.Mizuno); plzs3rog@okayama-u.ac.jp (H.Miyai); de19013@s.okayama-u.ac.jp (M.K.); mmorita@md.okayama-u.ac.jp (M.M.); 2 Advanced Research Center for Oral and Craniofacial Sciences, Okayama University Dental School, 2-5-1 Shikata-cho, Kita-ku, Okayama 700-8558, Japan; 3 Center for Innovative Clinical Medicine, Okayama University Hospital, 2-5-1 Shikata-cho, Kita-ku, Okayama 700-8558, Japan; E-Mail: t-maru@md.okayama-u.ac.jp

**Keywords:** microRNA, saliva, exosome, aging

## Abstract

The aim of this study was to examine whether salivary exosomal miRNAs could be identified as aging biomarkers. Fifteen young healthy volunteers (median age, 21.0 years) and 13 old individuals (median age, 66.0 years) were recruited. Unstimulated whole saliva was collected, salivary exosomes were isolated, and total RNA was extracted. In a microarray, 242 miRNAs were commonly detected in these two mixed samples. Based on the cut-off values of 2- or 0.5-fold changes (*FC*) and regulatory power for aging process, six candidate miRNAs (miR-24-3p, miR-371a-5p, miR-3175, miR-3162-5p, miR-671-5p, and miR-4667-5p) were selected. After comparing each total RNA obtained by the 15 young and 13 old individuals to validate the *FC* values using quantitative real-time PCR, miR-24-3p was identified as a novel candidate aging biomarker. This pilot study suggested that salivary exosomal miRNAs could be identified as candidate aging biomarkers. To confirm whether miR-24-3p in salivary exosomes are suitable biomarkers of aging, further validation research is required.

## 1. Introduction

Aging has been defined as a progressive organic functional decline with loss of homeostasis and increasing probability of illness and death [1]. All age-dependent diseases are connected to aging and these diseases have an influence on one another. Some of the mechanisms of aging have been revealed by modern biology [1,2], and the data suggest that passage of time alone is not the best measure of aging. Therefore, development of aging biomarkers is necessary to clearly define the aging and disease processes [3].

MicroRNAs (miRNAs) are single-stranded non-coding RNAs of approximately 21–23 nucleotides in length that can affect gene expression at the posttranscriptional level by binding to the three prime untranslated region (3′-UTR) of target messenger RNAs [4]. MiRNAs are present in several body fluids including amniotic fluid, breast milk, bronchial lavage, cerebrospinal fluid, colostrum, peritoneal fluid, plasma, pleural fluid, saliva, seminal fluid, tears and urine [5]. It has been reported that miRNAs are important regulators of cellular senescence and aging [6]. It is also known that serum miRNAs can act as diagnostic and prognostic biomarkers related to the biological and pathological processes of aging [7]. These data suggest that circulating miRNAs may be useful as aging biomarkers. However, there are few reports on the relationships between aging and miRNAs in body fluids other than blood.

Saliva contains a variety of molecular and microbial analytes, and may be an effective indicator of both local and systemic conditions [8]. It has been reported that blood-derived molecules entering salivary tissues via transcellular or paracellular routes affect the molecular composition of oral fluids [8]. It is also estimated that miRNA expression profiles in saliva are similar to those in serum [9]. Thus, miRNAs obtained in human saliva may also be considered as candidate aging biomarkers. In the present study, we hypothesized that salivary exosomal miRNAs are useful aging biomarkers. Therefore, the aim of this pilot study was to examine whether salivary exosomal miRNAs could be identified or not as aging biomarkers.

## 2. Results

### 2.1. Characteristics of Participants

Fifteen young participants and 14 old participants were recruited in this study. One old participant was excluded from the following analyses because the expression of internal control miRNA (miR-4739) was extremely low (raw threshold cycle (*C*_t_) value = 46.41). In advance, we set exclusion criteria to reduce the effects of systemic status on the miRNAs expression (see Methods). We included the participants with hypertension and dyslipidemia (two old participants), hyperuricemia (one old participant), and asthma (one young participant). We evaluated the characteristics including age, gender, body mass index (BMI), and oral conditions (Table 1). There were no significant differences in BMI, salivary flow rate, probing pocket depth (PPD), clinical attachment level (CAL) or bleeding on probing (BOP) between young and old groups. However, significant differences in number of teeth present (*p* < 0.001) and plaque control record (PCR) were observed (*p* = 0.016).

**Table 1:** Characteristics of young and old groups (median (25th, 75th percentile) or N (%)).

Variables	Categories	Young (N = 15)		Old (N = 13)		*p* Value
Age (years)	-	21.0	(21.0, 26.0)	66.0	(61.5, 72.5)	<0.001 *
Male (%)	-	8	(53.3)	6	(46.2)	0.705 ^†^
BMI	-	20.6	(20.1, 21.3)	22.4	(20.7, 24.3)	0.058 *
Salivary flow rate (ml/min)	-	0.32	(0.17, 0.90)	0.29	(0.17, 0.49)	0.363 *
Number of teeth presents	-	29.0	(28.0, 30.0)	27.0	(23.5, 28.0)	<0.001 *
PPD (mm)	-	2.04	(1.72, 2.10)	1.82	(1.66, 2.12)	0.496 *
CAL (mm)	-	2.04	(1.72, 2.10)	2.04	(1.71, 2.18)	0.717 *
BOP (%)	<20	11	(73.3)	12	(92.3)	0.333 ^‡^
-	≥20	4	(26.7)	1	(7.7)	-
PCR	<20	6	(40.0)	11	(84.6)	0.016 ^†^
-	≥20	9	(60.0)	2	(15.4)	-

* Mann-Whitney *U* test; ^†^ Chi-square test; ^‡^ Fischer’s exact test; N: number; BMI: body mass index; PPD: probing pocket depth; CAL: clinical attachment level; BOP: bleeding on probing; PCR: plaque control record.

### 2.2. Microarray Analysis and Candidate miRNA for Aging Biomarker

After normalization of the microarray data, 242 miRNAs were commonly detected in the two mixed total RNA samples obtained by five young and five old individuals, respectively (Table S1). We calculated the fold changes (*FC*) for the old group using the young group as reference. Using cut-off values of 2- or 0.5-*FC*, 61 miRNAs met this criteria and the novel out degree (NOD) values, which represents the number of uniquely regulated genes by one specific miRNA (see Methods), of these miRNAs were listed in Table 2. On the other hand, only 122 miRNAs of the commonly detected 242 miRNAs were entered in the DIANA database (Table S1). Based on the high (≥4) NOD values, cut-off values of 2- or 0.5-*FC*, and entry of DIANA database, miR-24-3p, miR-371a-5p, miR-3175, miR-3162-5p, miR-671-5p, and miR-4667-5p (the common prefix “hsa-” in the miRNA IDs were omitted) were considered as novel biomarker candidates for aging.

### 2.3. RT-qPCR Validation

**Figure 1:**
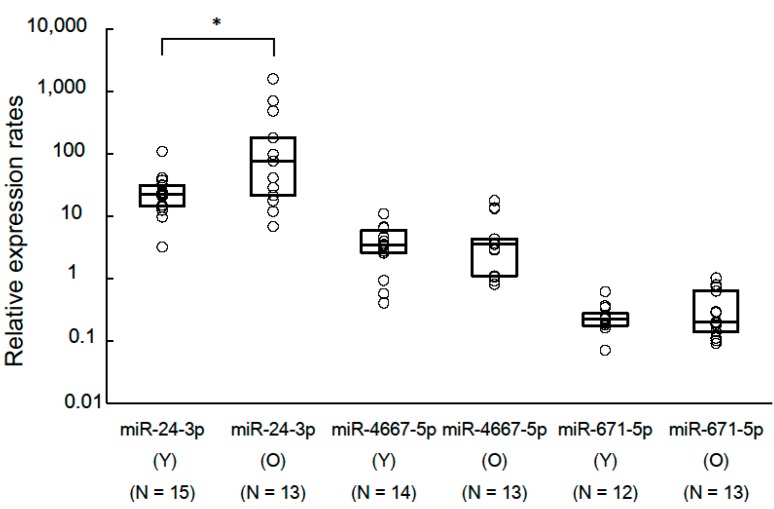
Comparison of relative expression rates of candidate miRNAs for aging biomarker between young and old groups. We performed TaqMan RT-qPCR to calculate the relative expression rates of three candidate miRNAs between young and old groups. We used miR-4739 as internal control miRNA. Circle plots represent relative expression rates of each sample. The horizontal lines within each box represent the 25, 50 and 75th percentiles. The *p* value was calculated by the Pair Wise Fixed Reallocation Randomisation Test using REST 2009 software. * *p* value < 0.05. N: number of samples. Y: samples of young group. O: samples of old group.

We selected miR-24-3p, miR-3175, miR-671-5p and miR-4667-5p for the candidate miRNAs from six miRNAs listed above because we could prepare the specific primers with sufficient sensitivity for these four miRNAs, which were contained by inventoried TaqMan miRNA assays (Applied Biosystems, Foster City, CA, USA). After we performed the quantitative real-time PCR (RT-qPCR) assays, miR-3175 was excluded from following analyses because 26 out of 28 (92.9%) samples had high (>35) raw *C*_t_ values. Using REST 2009 software (Qiagen, Valencia, CA, USA) and A Pair Wise Fixed Reallocation Randomisation Test, the relative expression rates (95% confidence interval, *p* value) of old group to young group of miR-24-3p, miR-4667-5p and miR-671-5p were 3.50 (0.19–115.37, *p* = 0.013), 1.12 (0.12–23.49, *p* = 0.774), and 1.18 (0.25–6.64, *p* = 0.563), respectively (Figure 1).

**Table 2:** List of the differentially expressed miRNAs between young and old groups.

(A) Increased Expression (*FC* > 2.0)			(B) Decreased Expression (*FC* < 0.5)		
miRNAs	*FC*	NOD *	miRNAs	*FC*	NOD *
miR-1343-3p	5.75	0	miR-1273e	0.19	0
miR-4725-3p	5.02	0	miR-7108-5p	0.24	0
miR-7106-5p	4.68	0	miR-6075	0.27	0
**miR-24-3p**	4.42	12	miR-4467	0.27	0
miR-6891-5p	4.08	0	miR-6127	0.28	0
miR-365a-5p	3.70	1	miR-1254	0.31	2
miR-6809-5p	3.65	0	miR-6828-5p	0.33	0
**miR-371a-5p**	3.58	15	miR-6859-5p	0.33	0
miR-6875-5p	3.55	0	miR-4486	0.38	1
miR-6824-5p	3.52	0	**miR-4667-5p**	0.41	7
miR-4534	3.46	2	miR-638	0.41	0
miR-8059	3.37	0	miR-8069	0.42	0
miR-6802-5p	3.18	0	miR-6724-5p	0.42	0
miR-3610	3.18	1	miR-6791-5p	0.42	0
**miR-3175**	3.14	4	miR-3180-3p	0.42	0
miR-7150	3.12	0	miR-4734	0.44	2
**miR-3162-5p**	3.11	5	miR-939-5p	0.44	0
miR-6800-5p	3.04	0	miR-3928-3p	0.44	0
miR-6768-5p	2.89	0	miR-1908-5p	0.46	0
miR-4722-5p	2.80	0	miR-4740-3p	0.46	0
miR-6131	2.75	0	miR-6729-5p	0.47	0
miR-6124	2.58	0	miR-3665	0.49	2
miR-6774-5p	2.35	0	miR-7846-3p	0.49	0
miR-6779-5p	2.32	0	miR-4787-5p	0.49	0
miR-6076	2.27	0	-	-	-
**miR-671-5p**	2.23	6	-	-	-
miR-4706	2.19	0	-	-	-
miR-7111-5p	2.16	0	-	-	-
miR-642b-3p	2.12	0	-	-	-
miR-128-2-5p	2.12	0	-	-	-
miR-1238-5p	2.11	0	-	-	-
miR-7847-3p	2.08	0	-	-	-
miR-1469	2.05	0	-	-	-
miR-7641	2.04	0	-	-	-
miR-6808-5p	2.04	0	-	-	-
miR-6726-5p	2.02	0	-	-	-
miR-4428	2.01	0	-	-	-

* NOD represents modified novel out degree that calculated by using commonly detected miRNAs and age-related pathways; *FC*: fold change of miRNAs for old group using young group as reference. The miRNAs with ≥4 NOD (novel out degree) are represented in bold.

### 2.4. Expression of miR-24-3p and Clinical Parameters

In old group, the relative expression of miR-24-3p was positively correlated with age (*p* = 0.042), PPD (*p* = 0.009), CAL (*p* = 0.010), and BOP (*p* = 0.025) and negatively correlated with salivary flow rate (*p* = 0.004) (Table 3). In young group, there were no associations between the expression of miR-24-3p and clinical parameters. In the old group, using multiple linear regression analysis, salivary flow rate (*p* = 0.028) and PPD (*p* = 0.013) were still significantly related with the relative expression of miR-24-3p (Table 4).

**Table 3:** Correlation between clinical parameters and relative expression rates for miR-24-3p.

Variables	Young (N = 15)		Old (N = 13)	
	*r* *	*p* Value	*r* *	*p* Value
Age (years)	−0.257	0.354	0.569	0.042
BMI (kg/m^2^)	−0.005	0.985	0.071	0.817
Salivary flow rate (mL/min)	0.191	0.494	−0.737	0.004
Number of teeth presents	−0.155	0.582	0.187	0.540
PPD (mm)	0.163	0.562	0.692	0.009
CAL (mm)	0.163	0.562	0.687	0.010
BOP (%)	−0.271	0.328	0.615	0.025
PCR (%)	−0.220	0.431	0.209	0.494

* Spearman’s rank correlation coefficient between each variable and relative expression rates for miR-24-3p using miR-4739 as reference; N: number; BMI: body mass index; PPD: probing pocket depth; CAL: clinical attachment level; BOP: bleeding on probing; PCR: plaque control record.

**Table 4:** Multiple linear regression analysis with the relative expression rates of miR-24-3p as the dependent variable in old group (N = 13).

Variables	B (95% CI)		β	*p* Value	VIF
Intercept	−1.41	(−9.19, 6.38)	-	0.696	-
Salivary flow rate (mL/min)	−5.52	(−10.32, −0.73)	−0.47	0.028	1.155
PPD (mm)	5.07	(1.33, 8.82)	0.55	0.013	1.155

We used stepwise method (entry, *p* = 0.05; removal, *p* = 0.1) to construct the model. The candidate variables for the model were age, salivary flow rate, PPD, CAL, and BOP (%). The final model was constructed based on the maximum adjusted R-squared and VIF less than 10. The *F*-statistic was 12.344 (*p* = 0.002), the R-squared was 0.712, and the adjusted R-squared was 0.654. N: number; CI: confidence interval; B: unstandardized regression coefficient; β: standardized coefficient; VIF: variance inflation factor; PPD: probing pocket depth; CAL: clinical attachment level; BOP: bleeding on probing.

### 2.5. Discrimination Power of miR-24-3p for Aging

**Figure 2:**
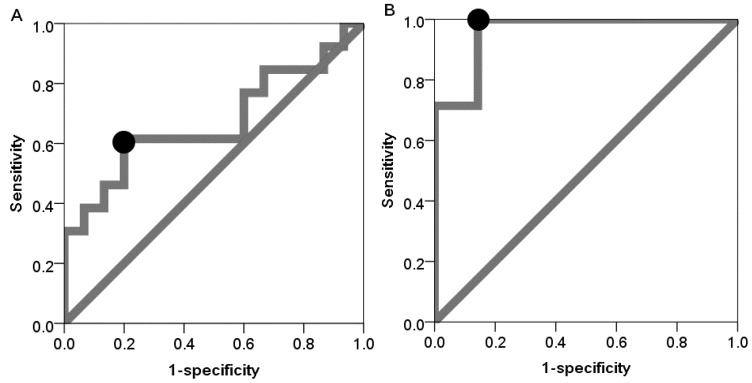

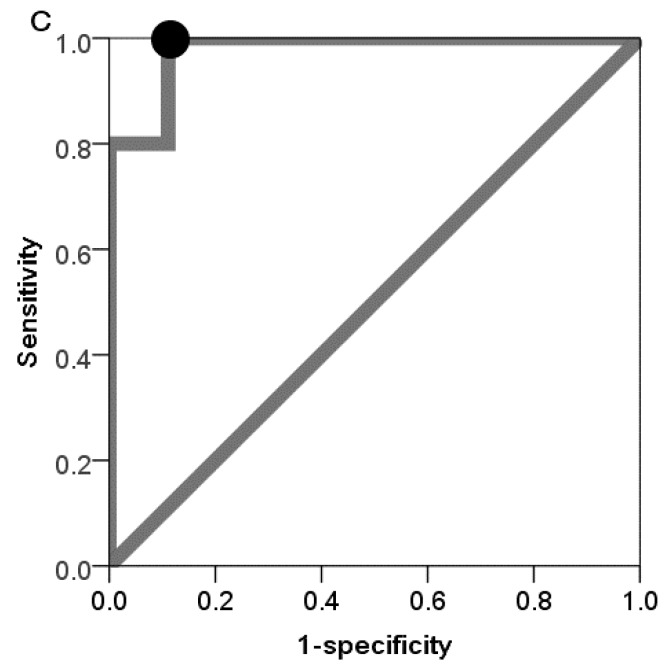
Receiver operating characteristic (ROC) curves for detection of old group in all participants (**A**); participants with low salivary flow rate (**B**); and participants with high probing pocket depth (**C**). The circle plots represent the sensitivity and specificity with optimal threshold value determined by the maximum Youden index (sensitivity + specificity − 1).

To evaluate the discrimination power of miR-24-3p for aging, receiver operating characteristic (ROC) curves were constructed for detection of old group (Figure 2). Because clinical parameter might affect the discrimination power, we constructed the ROC curves for all groups, the low salivary flow rate group, and the high PPD group based on the above results. Each of these latter two groups was categorized by median of salivary flow rate and PPD, respectively. In all groups, the results yielded an area under ROC curve (AUC) of 0.672 (*p* = 0.123) with a sensitivity and a specificity of 0.615 and 0.800, respectively. In the low salivary flow rate group, the results yielded an AUC of 0.959 (*p* = 0.004) with a sensitivity and a specificity of 1.000 and 0.857, respectively. In the high PPD group, the results yielded an AUC of 0.978 (*p* = 0.004) with a sensitivity and a specificity of 1.000 and 0.889, respectively (Table 5).

**Table 5:** Receiver operating characteristic curve analysis using expression rates of miR-24-3p to detect for old group.

Groups	Relative Expression Rates of miR-24-3p *		Young Group	Old Group	AUC	*p* Value ^†^	Sensitivity	Specificity
All group	High	(≥5.105)	3 (20.0)	8 (61.5)	0.672	0.123	0.615	0.800
	Low	(<5.105)	12 (80.0)	5 (38.5)	-	-	-	-
Low (<0.31(mL/min)) salivary flow rate group ^‡^	High	(≥4.930)	1 (14.3)	7 (100.0)	0.959	0.004	1.000	0.857
	Low	(<4.930)	6 (85.7)	0 (0.0)	-	-	-	-
High (≥2.01 (mm)) PPD group ^‡^	High	(≥5.805)	1 (11.1)	5 (100.0)	0.978	0.004	1.000	0.889
	Low	(<5.805)	8 (88.9)	0 (0.0)	-	-	-	-

* Relative expression rates of miR-24-3p normalized by log2 transformation were categorized by each optimal threshold value determined by the maximum Youden index (sensitivity + specificity − 1); ^†^ Nonparametric AUC estimation was conducted and the *p* value for testing AUC = 0.5 versus AUC ≠ 0.5 was calculated; ^‡^ Each of low salivary flow rate group and high PPD group was categorized by median of salivary flow rate and PPD, respectively. AUC: area under receiver operating characteristic curve. PPD: probing pocket depth.

### 2.6. Bioinformatics Analysis of miR-24-3p

We considered that *MYC*, *TCF7*, *IL1R1*, *TAB2*, *FASLG*, *TAOK1*, *FGF11*, *HSPA8*, *IL1A*, *SRF*, *RAP1B*, and *CRH* were unique (in 122 miRNAs set) target genes related to aging pathways for miR-24-3p (Table S2). The analysis showed that these genes were strongly associated with mitogen-activated protein kinase (MAPK) signaling pathway (*p* value corrected by Benjamini-Hochberg procedure was <0.001) (Table 6). The enrichment analysis indicated that miR-24-3p might regulate apoptotic processes, which could be associated with MAPK signaling pathway and pathways in cancer.

**Table 6:** Enrichment analysis for biological processes and pathways of unique target genes of miR-24-3p.

**(a) Annotation of Biological Processes**	**GO Term**	**Number of Related Genes**	*p* Value *	**B-H Test ^†^**
Response to organic substance	0010033	5	2.0 × 10^−3^	4.8 × 10^−1^
Positive regulation of multicellular organismal process	0051240	3	1.6 × 10^−2^	9.3 × 10^−1^
Immune response	0006955	4	1.6 × 10^−2^	8.3 × 10^−1^
Activation of pro-apoptotic gene products	0008633	2	1.8 × 10^−2^	7.7 × 10^−1^
Regulation of apoptosis	0042981	4	2.4 × 10^−2^	8.0 × 10^−1^
Regulation of programmed cell death	0043067	4	2.5 × 10^−2^	7.5 × 10^−1^
Regulation of cell death	0010941	4	2.5 × 10^−2^	7.0 × 10^−1^
Induction of apoptosis	0006915	3	2.7 × 10^−2^	6.8 × 10^−1^
Induction of programmed cell death	0012502	3	2.7 × 10^−2^	6.3 × 10^−1^
Regulation of cell cycle	0051726	3	2.8 × 10^−2^	6.2 × 10^−1^
Positive regulation of apoptosis	0043065	3	4.6 × 10^−2^	7.6 × 10^−1^
Positive regulation of programmed cell death	0043068	3	4.6 × 10^−2^	7.3 × 10^−1^
Positive regulation of cell death	0010942	3	4.7 × 10^−2^	7.1 × 10^−1^
Cell proliferation	0008283	3	4.7 × 10^−2^	6.8 × 10^−1^
**(b) KEGG Pathways**	**KEGG ID**	**Number of Related Genes**	***p* Value**	**B-H Test**
MAPK signaling pathway	hsa04010	10	1.3 × 10^−10^	5.6 × 10^−9^
Apoptosis	hsa04210	3	1.4 × 10^−2^	2.6 × 10^−1^
Pathways in cancer	hsa05200	4	3.0 × 10^−2^	3.4 × 10^−1^

The gene set including *MYC*, *TCF7*, *IL1R1*, *TAB2*, *FASLG*, *TAOK1*, *FGF11*, *HSPA8*, *IL1A*, *SRF*, *RAP1B*, and *CRH* was analyzed using DAVID online software. * The *p* value was calculated by DAVID online software with a modified Fisher’s Exact Test (EASE Score); ^†^ Benjamini-Hochberg multiple testing correction on the *p* values. GO: gene ontology.

## 3. Discussion

To the best of our knowledge, this is the first report to investigate possible aging biomarkers obtained from salivary exosomal miRNAs. The analyses revealed that miR-24-3p was identified as a novel candidate aging biomarker. Moreover, oral conditions could affect the discrimination power of miR-24-3p. Especially in the low salivary flow rate group and high PPD group, using the expression rates of miR-24-3p could correctly discriminate the old group from the young group. This pilot study indicated that salivary exosomal miRNAs could serve as aging biomarkers.

Aging is correlated with increased prevalence and severity of periodontitis [10]. In this study, we recruited the participants without severe periodontitis. However, there were statistically significant correlations between miR-24-3p and oral conditions in old participants. It is feasible that miR-24-3p could reflect periodontal inflammation at a subclinical level in the elderly.

Inflamm-aging, which is chronic low-grade inflammation during aging, is commonly characterized by pro-inflammatory biomarkers [11]. Our enrichment analysis indicated that the unique target genes of miR-24-3p could strongly affect MAPK signaling pathway, which is involved in inflammatory cytokine and chemokine gene regulation at both the transcriptional and the post-transcriptional levels [12]. The increase in miR-24-3p may contribute to increased susceptibility to age-dependent alterations in the immune and inflammatory status.

The present result also showed that expression rate of salivary miR-24-3p was negatively correlated with salivary flow rate in old participants but not in young participants. This suggests that expression rates of salivary miR-24-3p reflect age-related decline in salivary function. In our findings, target genes of miR-24-3p might be associated with apoptosis. It has been reported that an increased number of apoptotic salivary epithelial cells may contribute to the decreased saliva production and excretion in aging mouse [13]. It is suggested that miR-24-3p could induce apoptosis of salivary glands during aging, and such conditions might decrease salivary functions in the elderly. On the other hand, exosomes are released from many cell types including dendritic cells, lymphocytes, platelets mast cells, epithelial cells, endothelial cells, and neurons, and can be found in several types of body fluids including saliva [14]. It is still unclear which cell types released miR-24-3p and affect whether systemic aging and/or tissue-specific aging including oral tissue and salivary gland. Further studies are needed to clarify this point.

The unique target genes and modified NOD value could be varied with the set of exosomal miRNAs. Moreover, all of the target genes could be related to aging processes. Therefore, to clarify the more detailed function of miR-24-3p for aging processes, regulatory power of miR-24-3p should be analyzed with the different set of exosomal miRNAs obtained by different body fluids or tissues in the future study.

The reasons why miR-4739 was considered as suitable for internal control miRNA were as follows. In samples of 15 young participants and 13 old participants, miR-4739 represented consistent expression (raw *C*_t_ ≤ 35). Mann-Whitney *U* test indicated that raw *C*_t_ value of miR-4739 were stable in young and old groups (*p* = 0.339). Moreover, the expression of miR-4739 and the amounts of total RNA were significantly correlated with Spearman’s rank correlation test (*r* = 0.450, *p* = 0.016).

Studies have investigated the relationships between miRNA profiles and aging. For instance, a clinical study reported that serum expression levels of miR-151a-3p, miR-181a-5p and miR-1248 were significantly lower in old participants (mean age, 64 years) than in young participants (mean age, 30 years) [15]. Another clinical study using serum samples also showed that PCR analysis identified five down-regulated miRNAs (miR-29b, miR-106b, miR-130b, miR-142-5p and miR-340) and three up-regulated miRNAs (miR-92a, miR-222 and miR-375) with age [7]. Among our findings, miR-24-3p in saliva was confirmed to show significant age-related increases. The present and previous findings support the notion that circulating miRNAs are useful for aging biomarkers. However, in our study, overlap of the relative expression profile of miR-24-3p were also found in the young and the old groups. This suggests that miR-24-3p might be a non-specific screening biomarker for aging.

Expression of miRNA profiles in saliva may be similar to those in serum. However, the results of the present and previous studies indicate that there seems to be little overlap of age-associated miRNAs between serum and saliva. The miRNAs in saliva could be produced by components of the salivary gland. Therefore, our results may more reflect the aging change of the salivary gland than a whole body.

The limitations of this pilot study are as follows. External validity is limited because all participants were recruited at the Department of Preventive Dentistry, Okayama University Hospital. Correlation between expression level of miR-24-3p and age are still unclear because the participants did not cover wide range of age and this study design was cross-sectional. Moreover, subclinical status might affect these results especially in the old group.

## 4. Experimental Section

### 4.1. Study Participants

This study was in accordance with the Declaration of Helsinki [16] and was approved by the Ethics Committee of Okayama University Hospital (approval number: 1975). After obtaining informed consent, participants who fulfilled the study requirements were enrolled. We considered that the minimum sample size for this pilot study was 12 per group, because there was no prior information to base a sample size on [17]. Fifteen young participants (median age, 21.0 years) and 13 old participants (median age, 66.0 years) were finally recruited at the Department of Preventive Dentistry, Okayama University Hospital from August 2014 to December 2014. Exclusion criteria were as follows: cancer; diabetes; chronic lung disease other than asthma; cardiovascular event in the past six months; renal dysfunction; hepatic dysfunction; dementia; severe immune alterations; autoimmune diseases; current smoker; and pregnancy at the time of saliva collection. All of these were as reported in previous studies related to aging [18,19,20]. In addition, severe periodontitis [21] was set as an exclusion criterion.

### 4.2. Questionnaire

Participants filled out the self-administered questionnaire for assessment of systemic conditions. Weight and height of participants were also recorded and BMI (weight in kilograms per square of height in meters) was calculated for each participant.

### 4.3. Oral Examination

PPD and CAL were determined at six sites (mesio-buccal, mid-buccal, disto-buccal, mesio-lingual, mid-lingual and disto-lingual) on all teeth using a color-coded probe (Hu-Friedy; Chicago, IL, USA). Sites that bled upon gentle probing (25 g probing force) were recorded, and the proportion of sites with BOP was measured. PCR were measured after staining with erythrosine, and were recorded in terms of presence or absence adjacent to the gingival margin at four sites (mesial, distal, buccal and lingual) around each tooth [22]. All clinical procedures were performed by four trained dentists (Takaaki Tomofuji, Takayuki Maruyama, Tatsuya Machida, and Yuya Kawabata).

### 4.4. Saliva Collection

Unstimulated whole saliva was collected as reported previously with minor modification [23]. Briefly, saliva was collected in the morning (7:00 AM to 12:00 noon). During the collection period, participants were seated straight up and were instructed to refrain from speaking or swallowing. They allowed the saliva to accumulate in the floor of the mouth, and then spit it through a funnel into a tube kept on ice. At least 1 mL of unstimulated whole saliva was collected. Salivary flow rate was calculated by dividing the volume of collected saliva by the duration of collection time. After collection of saliva, it was stored at 4 °C for up to 6 h, after which it was stored at −80 °C until use.

### 4.5. Exosome Isolation

Exosomes were isolated from saliva samples (0.5–1.0 mL) using total exosome isolation reagent (Invitrogen, Carlsbad, CA, USA), in accordance with the manufacturer’s protocol. Briefly, whole saliva was centrifuged at 2000× *g* (*g* indicates gravity) for 10 min at room temperature to remove cells and debris. Supernatant was incubated with total exosome isolation reagent at 2 to 8 °C for 1 h. After incubation, samples were centrifuged at 10,000× *g* for 1 h at 2 to 8 °C. Residual supernatant was then discarded and the exosome pellet was collected. The pellet was then re-suspended in a 0.1 volume (calculated by salivary supernatant volume) of exosome re-suspension buffer (Invitrogen).

### 4.6. RNA Extraction

Total exosome RNA and protein isolation kits (Invitrogen) were utilized for extraction of total RNA from exosome samples. The exosome pellet sample was incubated for 5–10 min at room temperature in order to allow the pellet to dissolve and 200 μL of each sample (brought up to volume with exosome re-suspension buffer if necessary) was combined with 200 μL of 2× denaturing solution, and was incubated on ice for 5 min. After incubation, 400 μL of acid-phenol chloroform was added to the mixture, followed by vortexing for 30–60 s. Samples were centrifuged for 5 min at 10,000× *g* at room temperature to separate the mixture into the aqueous (upper) phase, interphase and lower phase. The 250 μL of aqueous phase, which contains total RNA, was removed and transferred into a new tube. Then, 99.5% ethanol was added to the aqueous phase, up to 700 μL was placed onto a spin column in a collection tube, and it was centrifuged at 10,000× *g* for 15 s. After discarding the flow-through and saving the spin column, the sample was washed three times with wash solution mixed with 99.5% ethanol (centrifuged for 15 s for each wash). After washing, the filter was dried by a further 1 min centrifuging at 10,000× *g* and the filter cartridge was transferred into a fresh collection tube. Next, 100 μL of preheated (95 °C) nuclease-free water was applied to the center of the filter and the sample was centrifuged for 30 s at 10,000× *g* to recover total RNA. We repeated this procedure three times and the elution containing the RNA was collected. It was stored at 4 °C for up to 6 h, after which it was stored at −80 °C until use. After extraction of total RNA from salivary exosome, we confirmed the quality of them using the Agilent 2100 bioanalyzer (Agilent Technologies, Carlsbad, CA, USA) and the Agilent RNA 6000 Pico Kit (Agilent Technologies).

### 4.7. Microarray Analysis

A microarray analysis using 3D-Gene (Toray, Kanagawa, Japan) was performed. We prepared two pooled RNA samples by mixing equal amounts (20 ng) of total RNA, which were calculated using the Agilent 2100 bioanalyzer (Agilent Technologies), obtained by five young and five old participants, respectively. These samples had at least 100 pg/μL of total RNA and were mostly consisted of small RNA (less than 500 nucleotide). Samples were labeled with the 3D-Gene miRNA labeling kit (Toray) and hybridized onto 3D-Gene human miRNA Oligo chips v20 in accordance with the supplier’s protocols (http://www.3d-gene.com). Fluorescent signals were scanned with the 3D-Gene Scanner and analyzed using 3D-Gene Extraction software (Toray). Raw data for each spot was normalized by substitution with a mean intensity for background signals, which were determined from blank spots’ signal intensities of 95% confidence intervals. Measurement of spots with signal intensities greater than 2 standard deviations (SD) from the background signal intensity were considered to be valid. Background-subtracted signal intensities were calculated for these spots. We used the global normalization method (ratio median = 1). We calculated *C*_t_ values of old samples for each miRNA using the signals of young samples as reference.

### 4.8. Selecting Candidate miRNAs for Aging Biomarkers

We used the DIANA miRPath v.2.0. [24] to predict target genes and pathways of the miRNAs. To evaluate the regulatory power involved in aging processes, we calculated the novel out degree (NOD) values [25] with modification. NOD values have been proposed to measure the independent regulatory power of individual miRNAs. In other words, NOD values represents the number of uniquely regulated genes by one specific miRNA [25]. We considered the miRNAs with high regulatory power, which means the miRNAs could uniquely (in our miRNAs set) regulate many target genes involved in aging processes, as suitable for candidate aging biomarkers. We also considered the miRNAs with NOD values (≥4) [25] as miRNAs with high regulatory power for aging. Several research groups have performed genome-wide analyses of transcriptional profiling of aging in human tissues, including brain, kidney, muscle, skin and blood. Common aging-related signature genes and the Kyoto Encyclopedia of Genes and Genomes (KEGG) pathways [26] related to these genes were listed in a previous study [27]. To focus on regulatory power for the aging process, we calculated NOD values using only genes related to these aging pathways. Moreover, the miRNAs commonly detected in microarray and could be analyzed in DIANA miRPath were used to calculate NOD values. These list of miRNAs was uploaded to the online software. The default DIANA-microT-CDS cut-off value of 0.8 was applied for filtering of miRNA-gene interactions. To increase the strictness of the target pathway prediction, a posteriori approach (pathway union) was applied. miRNA and pathway related information was obtained from miRBase 18 and KEGG v58.1, respectively, for homo sapiens.

### 4.9. RT-qPCR

To validate the microarray data, we performed TaqMan RT-qPCR assays. We used the inventoried TaqMan microRNA Assays (Life Technologies, Carlsbad, CA, USA) for the RT-qPCR analyses performed on Mx3000P Real-time QPCR System (Agilent Technologies) according to the manufacturer’s instructions [28,29]. Briefly, reverse transcription (RT) enzymes including specific RT primers for each miRNA and total RNA sample were mixed in a tube. RT run conditions were then set. The plate was incubated at 16 °C for 30 min, 42 °C for 30 min and 85 °C for 5 min, and was then held at 4 °C. Products of the RT reaction were stored at −20 °C until use. RT-qPCR was performed in triplicate using TaqMan Universal Master Mix II, no UNG (Applied Biosystems, Life Technologies, Carlsbad, CA, USA). After holding at 95 °C for 10 min, fifty thermal cycles (95 °C for 15 s and 60 °C for 60 s) were run. *C*_t_ values were determined using background-based threshold (cycle-range 5 to 8) calculated by the MxPro Mx3000P v4.10 software (Stratagene, Edinburgh, UK). Data with a >35 raw *C*_t_ value for each miRNA was excluded from each analysis. We considered the miRNAs with consistent expression in our study as suitable for internal control miRNA because internal control small RNAs for normalization of salivary exosomal miRNAs have not yet clearly decided [23]. In addition, we considered that the miRNAs with high NOD value, which indicates high regulatory power in aging process, could serve as useful internal control miRNAs. This is based on the concept that identification of multiple miRNA changes could be valuable because specific characteristics of miRNA combinations unique to a normal physiological or pathological state could serve as useful reference [30]. In our study, miR-4739 was considered as suitable for internal control miRNAs. We calculated the relative expression rates of each miRNA between young and old groups using 2^−∆∆*C*t^ method [31]. To determine the statistical significance of relative expression levels of each miRNA between young and old groups, we performed the bootstrapping approach (Pair Wise Fixed Reallocation Randomisation Test) using REST 2009 software with 2000 random reallocations of samples [32]. This statistical test could be suitable for our analysis because it could avoid making any assumptions about distributions [32].

### 4.10. Relationships of miRNA and Clinical Parameters

It has been reported that periodontitis and obesity affect the expression of circulating miRNAs [33]. Therefore, we analyzed the relationships between candidate miRNA and clinical parameters including age, BMI, and oral conditions in young and old groups using Spearman’s rank correlation test. Furthermore, we analyzed the relationship between candidate miRNA and significantly correlated variables using multiple linear regression analysis with stepwise method. The relative expression of candidate miRNA was normalized by log2 transformation for the analysis.

### 4.11. Discrimination Power of Candidate miRNA Biomarker for Aging

To evaluate the discrimination power of candidate miRNA biomarker for aging, ROC curves [34] were constructed for detection of old group. The ROC curves were obtained by plots of the sensitivity (true positive rate) and 1-specificity (false positive rate) of the diagnostic test at various cut-off values. The ROC curves graphically show the relationship between both the true positive rate and false positive rate according to the various cut-off values, which are effective to determine the optimal cut-off values. AUC summarizes the entire location of the ROC curve and reflects the discrimination power of the biomarkers. To decide the optimal threshold value, we used the Youden index (sensitivity + specificity − 1) [35].

### 4.12. Enrichment Analysis for Biological Process and Pathway of Target Genes

An enrichment analysis for biological process and pathway of target genes was performed using the Database for Annotation, Visualization and Integrated Discovery (DAVID) v6.7 (http://david.abcc.ncifcrf.gov) [36] using the homosapiens background list. It was pointed out that multiple testing correction techniques are conservative approaches and it could hurt the sensitivity of discovery if overemphasizing them [36]. Therefore, we considered that the gene ontology (GO) terms and KEGG pathways with *p* value (without multiple testing correction) <0.05 also warranted discussion (even though their corrected *p* value was not significant).

### 4.13. Statistical Analyses

Mann-Whitney *U* test, chi-square test and Fischer’s exact test were used for comparison of the characteristics of young and old groups. Values of two-sided *p* < 0.05 were considered to represent statistically significant differences. These statistical analyses were performed using SPSS software version 20 (SPSS Inc., Chicago, IL, USA).

## 5. Conclusions

miR-24-3p extracted by salivary exosomes may serve as aging biomarkers. Saliva is one of the most accessible and noninvasive body fluids. Detecting salivary exosomal miRNAs may provide a simple means for measuring aging and assessing new findings to understand the aging process. Further validation research is required to overcome the limitations listed above and confirm whether miR-24-3p in salivary exosome are suitable biomarkers of aging.

## Supplementary Materials

Supplementary materials can be found at http://www.mdpi.com/1422-0067/16/09/21294/s1.
