# Supplementary material for: MicroRNAs in Salivary Exosome as Potential Biomarkers of Aging
Source: Int J Mol Sci. 2015 Sep 7;16(9):21294–309. doi: 10.3390/ijms160921294 (PMC4613253; doi:10.3390/ijms160921294)
Supplement: Supplementary file 1 [file ijms-16-21294-s001.pdf]

Supplementary Information

Table S1. List of commonly detected miRNAs in young and old groups using microarray.

miRNA Name	miRBase Mature miRNA ID	Signal Intensity *		FC †	Entry of DIANA Database ‡
		Young Group	Old Group		
miR-24-3p	MIMAT0000080	5.83	25.75	4.42	Entried
miR-198	MIMAT0000228	11.77	13.24	1.13	Entried
miR-203a	MIMAT0000264	21.48	11.73	0.55	Not entried
miR-124-3p	MIMAT0000422	6.12	11.43	1.87	Entried
miR-370-3p	MIMAT0000722	8.31	7.21	0.87	Not entried
miR-491-5p	MIMAT0002807	5.32	6.19	1.16	Entried
miR-638	MIMAT0003308	234.16	96.25	0.41	Entried
miR-663a	MIMAT0003326	94.41	60.46	0.64	Entried
miR-658	MIMAT0003336	4.64	9.16	1.97	Entried
miR-671-5p	MIMAT0003880	9.68	21.63	2.23	Entried
miR-30c-2-3p	MIMAT0004550	6.63	5.00	0.75	Entried
miR-149-3p	MIMAT0004609	52.07	51.31	0.99	Entried
miR-371a-5p	MIMAT0004687	4.79	17.15	3.58	Entried
miR-92b-5p	MIMAT0004792	12.79	19.52	1.53	Entried
miR-551b-5p	MIMAT0004794	197.93	213.40	1.08	Entried
miR-744-5p	MIMAT0004945	8.28	7.36	0.89	Entried
miR-920	MIMAT0004970	67.52	55.91	0.83	Entried
miR-939-5p	MIMAT0004982	121.49	53.73	0.44	Not entried
miR-1224-5p	MIMAT0005458	8.49	12.40	1.46	Entried
miR-1228-5p	MIMAT0005582	63.94	46.61	0.73	Entried
miR-1290	MIMAT0005880	58.36	32.52	0.56	Entried
miR-1246	MIMAT0005898	152.10	121.90	0.80	Entried
miR-1254	MIMAT0005905	174.89	54.34	0.31	Entried
miR-1268a	MIMAT0005922	55.69	46.80	0.84	Entried
miR-1469	MIMAT0007347	18.37	37.67	2.05	Entried
miR-1908-5p	MIMAT0007881	153.55	69.99	0.46	Not entried
miR-1914-3p	MIMAT0007890	17.35	22.87	1.32	Entried
miR-1915-3p	MIMAT0007892	40.16	20.52	0.51	Entried
miR-365a-5p	MIMAT0009199	4.79	17.72	3.70	Entried
miR-762	MIMAT0010313	143.41	112.38	0.78	Entried
miR-711	MIMAT0012734	33.87	19.51	0.58	Entried

Table S1. *Cont*

miRNA Name	miRBase Mature miRNA ID	Signal Intensity *		FC †	Entry of DIANA Database ‡
		Young Group	Old Group		
miR-2861	MIMAT0013802	162.58	102.04	0.63	Entried
miR-3131	MIMAT0014996	113.90	57.53	0.51	Entried
miR-3162-5p	MIMAT0015036	4.89	15.22	3.11	Entried
miR-1193	MIMAT0015049	9.83	5.96	0.61	Entried
miR-3175	MIMAT0015052	5.42	17.03	3.14	Entried
miR-3178	MIMAT0015055	47.61	58.71	1.23	Entried
miR-3180-3p	MIMAT0015058	46.94	19.93	0.42	Entried
miR-3185	MIMAT0015065	13.74	14.01	1.02	Entried
miR-3188	MIMAT0015070	9.33	13.10	1.40	Entried
miR-3196	MIMAT0015080	127.15	70.48	0.55	Entried
miR-3197	MIMAT0015082	56.01	60.75	1.08	Entried
miR-4294	MIMAT0016849	33.38	61.49	1.84	Entried
miR-4299	MIMAT0016851	7.18	12.46	1.74	Entried
miR-4257	MIMAT0016878	24.67	21.44	0.87	Entried
miR-4260	MIMAT0016881	28.01	39.75	1.42	Entried
miR-4327	MIMAT0016889	12.98	16.62	1.28	Entried
miR-4270	MIMAT0016900	17.37	23.53	1.35	Entried
miR-4271	MIMAT0016901	27.57	36.25	1.31	Entried
miR-4281	MIMAT0016907	53.49	50.30	0.94	Entried
miR-3610	MIMAT0017987	9.80	31.16	3.18	Entried
miR-3621	MIMAT0018002	80.58	85.70	1.06	Entried
miR-3648	MIMAT0018068	678.78	359.98	0.53	Entried
miR-3652	MIMAT0018072	7.86	7.08	0.90	Entried
miR-3656	MIMAT0018076	130.15	75.00	0.58	Entried
miR-1273e	MIMAT0018079	7.34	1.41	0.19	Entried
miR-3665	MIMAT0018087	338.65	165.65	0.49	Entried
miR-3679-5p	MIMAT0018104	13.91	25.30	1.82	Entried
miR-3180	MIMAT0018178	34.50	17.74	0.51	Entried
miR-3928-3p	MIMAT0018205	159.38	70.84	0.44	Not entried
miR-3937	MIMAT0018352	18.90	15.96	0.84	Entried
miR-642b-3p	MIMAT0018444	14.90	31.59	2.12	Entried
miR-1268b	MIMAT0018925	38.62	34.34	0.89	Entried
miR-4428	MIMAT0018943	5.88	11.82	2.01	Entried
miR-4429	MIMAT0018944	5.44	4.31	0.79	Entried
miR-4430	MIMAT0018945	7.53	7.86	1.04	Entried
miR-4442	MIMAT0018960	86.44	77.15	0.89	Entried
miR-4454	MIMAT0018976	25.84	15.86	0.61	Entried
miR-4459	MIMAT0018981	61.24	69.56	1.14	Entried
miR-3135b	MIMAT0018985	6.94	8.49	1.22	Entried
miR-4463	MIMAT0018987	51.58	53.32	1.03	Entried
miR-4466	MIMAT0018993	168.70	121.62	0.72	Entried
miR-4467	MIMAT0018994	201.04	54.96	0.27	Entried
miR-4478	MIMAT0019006	8.74	5.48	0.63	Entried

Table S1. *Cont*

miRNA Name	miRBase Mature miRNA ID	Signal Intensity *		FC †	Entry of DIANA Database ‡
		Young Group	Old Group		
miR-4484	MIMAT0019018	381.51	252.60	0.66	Entried
miR-4486	MIMAT0019020	17.40	6.62	0.38	Entried
miR-4488	MIMAT0019022	236.24	123.14	0.52	Entried
miR-4492	MIMAT0019027	19.40	14.00	0.72	Entried
miR-4497	MIMAT0019032	68.55	100.48	1.47	Entried
miR-4419b	MIMAT0019034	46.49	27.04	0.58	Entried
miR-4505	MIMAT0019041	45.57	22.99	0.50	Entried
miR-4508	MIMAT0019045	188.24	104.93	0.56	Entried
miR-4513	MIMAT0019050	14.21	9.41	0.66	Entried
miR-4516	MIMAT0019053	211.72	175.83	0.83	Entried
miR-4530	MIMAT0019069	131.63	189.67	1.44	Entried
miR-4532	MIMAT0019071	15.71	23.54	1.50	Entried
miR-4534	MIMAT0019073	15.85	54.83	3.46	Entried
miR-3619-3p	MIMAT0019219	19.08	21.26	1.11	Entried
miR-3940-5p	MIMAT0019229	104.79	54.20	0.52	Entried
miR-3960	MIMAT0019337	707.86	424.37	0.60	Entried
miR-4646-5p	MIMAT0019707	6.33	4.72	0.75	Entried
miR-4649-5p	MIMAT0019711	8.49	11.79	1.39	Entried
miR-4651	MIMAT0019715	34.71	33.69	0.97	Entried
miR-4655-5p	MIMAT0019721	33.67	37.05	1.10	Entried
miR-4656	MIMAT0019723	19.96	15.11	0.76	Entried
miR-4665-5p	MIMAT0019739	23.20	20.91	0.90	Entried
miR-4667-5p	MIMAT0019743	83.96	34.46	0.41	Entried
miR-4674	MIMAT0019756	5.64	9.37	1.66	Entried
miR-4687-3p	MIMAT0019775	19.69	30.01	1.52	Entried
miR-1343-3p	MIMAT0019776	4.93	28.38	5.75	Not entried
miR-4688	MIMAT0019777	32.73	56.91	1.74	Entried
miR-4689	MIMAT0019778	56.81	97.87	1.72	Entried
miR-4695-5p	MIMAT0019788	65.79	54.50	0.83	Entried
miR-4706	MIMAT0019806	13.00	28.49	2.19	Entried
miR-4722-5p	MIMAT0019836	14.08	39.47	2.80	Entried
miR-4723-5p	MIMAT0019838	988.71	678.22	0.69	Entried
miR-4725-3p	MIMAT0019844	6.15	30.84	5.02	Entried
miR-4726-5p	MIMAT0019845	27.18	23.29	0.86	Entried
miR-4728-5p	MIMAT0019849	36.39	27.01	0.74	Entried
miR-4730	MIMAT0019852	38.17	49.19	1.29	Entried
miR-4732-5p	MIMAT0019855	33.59	24.51	0.73	Entried
miR-4734	MIMAT0019859	107.17	46.72	0.44	Entried
miR-4739	MIMAT0019868	94.52	111.66	1.18	Entried
miR-4740-3p	MIMAT0019870	50.38	23.14	0.46	Entried
miR-4741	MIMAT0019871	37.18	36.45	0.98	Entried
miR-4745-5p	MIMAT0019878	26.82	26.82	1.00	Entried
miR-4749-5p	MIMAT0019885	25.25	20.09	0.80	Entried

Table S1. *Cont*

miRNA Name	miRBase Mature miRNA ID	Signal Intensity *		FC †	Entry of DIANA Database ‡
		Young Group	Old Group		
miR-4751	MIMAT0019888	10.84	9.77	0.90	Entried
miR-4758-5p	MIMAT0019903	15.26	13.84	0.91	Entried
miR-4763-3p	MIMAT0019913	27.38	21.23	0.78	Entried
miR-4787-5p	MIMAT0019956	308.93	152.58	0.49	Entried
miR-642a-3p	MIMAT0020924	18.97	33.49	1.77	Entried
miR-5001-5p	MIMAT0021021	70.82	50.09	0.71	Entried
miR-5008-5p	MIMAT0021039	21.16	39.22	1.85	Entried
miR-197-5p	MIMAT0022691	74.11	73.38	0.99	Entried
miR-204-3p	MIMAT0022693	139.17	267.37	1.92	Entried
miR-211-3p	MIMAT0022694	8.04	7.62	0.95	Entried
miR-345-3p	MIMAT0022698	5.08	7.47	1.47	Entried
miR-1227-5p	MIMAT0022941	37.98	24.86	0.65	Not entried
miR-1229-5p	MIMAT0022942	20.64	25.31	1.23	Not entried
miR-1233-5p	MIMAT0022943	532.98	766.18	1.44	Not entried
miR-1237-5p	MIMAT0022946	172.81	143.85	0.83	Not entried
miR-1238-5p	MIMAT0022947	19.32	40.70	2.11	Not entried
miR-4632-5p	MIMAT0022977	22.00	24.63	1.12	Not entried
miR-5739	MIMAT0023116	20.52	16.37	0.80	Not entried
miR-5787	MIMAT0023252	241.70	273.71	1.13	Not entried
miR-6075	MIMAT0023700	50.25	13.46	0.27	Not entried
miR-6076	MIMAT0023701	14.44	32.78	2.27	Not entried
miR-6085	MIMAT0023710	187.28	140.96	0.75	Not entried
miR-6087	MIMAT0023712	97.21	64.03	0.66	Not entried
miR-6088	MIMAT0023713	32.42	32.43	1.00	Not entried
miR-6089	MIMAT0023714	380.64	243.43	0.64	Not entried
miR-6090	MIMAT0023715	298.99	192.58	0.64	Not entried
miR-6124	MIMAT0024597	8.21	21.22	2.58	Not entried
miR-6125	MIMAT0024598	360.58	231.97	0.64	Not entried
miR-6126	MIMAT0024599	170.06	106.28	0.62	Not entried
miR-6127	MIMAT0024610	15.54	4.37	0.28	Not entried
miR-6131	MIMAT0024615	20.12	55.31	2.75	Not entried
miR-6132	MIMAT0024616	12.55	10.01	0.80	Not entried
miR-6165	MIMAT0024782	5.00	8.52	1.71	Not entried
miR-6721-5p	MIMAT0025852	8.75	13.43	1.54	Not entried
miR-6722-3p	MIMAT0025854	17.69	16.88	0.95	Not entried
miR-6724-5p	MIMAT0025856	46.75	19.67	0.42	Not entried
miR-328-5p	MIMAT0026486	72.67	94.63	1.30	Not entried
miR-1343-5p	MIMAT0027038	49.66	48.68	0.98	Not entried
miR-6726-5p	MIMAT0027353	13.55	27.34	2.02	Not entried
miR-6727-5p	MIMAT0027355	157.20	93.40	0.59	Not entried
miR-6728-5p	MIMAT0027357	14.28	20.86	1.46	Not entried
miR-6729-5p	MIMAT0027359	203.77	95.27	0.47	Not entried
miR-6732-5p	MIMAT0027365	12.77	10.59	0.83	Not entried

Table S1. *Cont*

miRNA Name	miRBase Mature miRNA ID	Signal Intensity *		FC †	Entry of DIANA Database ‡
		Young Group	Old Group		
miR-6738-5p	MIMAT0027377	7.56	13.39	1.77	Not entried
miR-6741-5p	MIMAT0027383	24.43	24.56	1.01	Not entried
miR-6743-5p	MIMAT0027387	50.25	77.23	1.54	Not entried
miR-6746-5p	MIMAT0027392	136.80	122.13	0.89	Not entried
miR-6749-5p	MIMAT0027398	41.75	44.24	1.06	Not entried
miR-6752-5p	MIMAT0027404	56.38	88.99	1.58	Not entried
miR-6756-5p	MIMAT0027412	38.02	48.97	1.29	Not entried
miR-6757-5p	MIMAT0027414	91.65	125.45	1.37	Not entried
miR-6760-5p	MIMAT0027420	70.69	75.07	1.06	Not entried
miR-6763-5p	MIMAT0027426	5.37	7.51	1.40	Not entried
miR-6765-5p	MIMAT0027430	35.27	28.00	0.79	Not entried
miR-6767-5p	MIMAT0027434	6.06	6.87	1.13	Not entried
miR-6768-5p	MIMAT0027436	6.39	18.48	2.89	Not entried
miR-6769a-5p	MIMAT0027438	79.16	56.10	0.71	Not entried
miR-6771-5p	MIMAT0027442	38.75	49.97	1.29	Not entried
miR-6772-5p	MIMAT0027444	29.86	15.68	0.53	Not entried
miR-6774-5p	MIMAT0027448	22.92	53.93	2.35	Not entried
miR-6775-5p	MIMAT0027450	17.57	28.32	1.61	Not entried
miR-6777-5p	MIMAT0027454	14.44	10.26	0.71	Not entried
miR-6778-5p	MIMAT0027456	42.64	30.54	0.72	Not entried
miR-6779-5p	MIMAT0027458	10.58	24.52	2.32	Not entried
miR-6781-5p	MIMAT0027462	54.67	40.39	0.74	Not entried
miR-6782-5p	MIMAT0027464	8.30	14.81	1.78	Not entried
miR-6784-5p	MIMAT0027468	62.34	62.27	1.00	Not entried
miR-6785-5p	MIMAT0027470	16.72	29.38	1.76	Not entried
miR-6786-5p	MIMAT0027472	242.26	137.41	0.57	Not entried
miR-6787-5p	MIMAT0027474	77.35	57.99	0.75	Not entried
miR-6789-5p	MIMAT0027478	9.33	10.99	1.18	Not entried
miR-6791-5p	MIMAT0027482	77.60	32.82	0.42	Not entried
miR-6794-5p	MIMAT0027488	14.45	17.40	1.20	Not entried
miR-6798-5p	MIMAT0027496	30.92	46.08	1.49	Not entried
miR-6799-5p	MIMAT0027498	9.81	18.46	1.88	Not entried
miR-6800-5p	MIMAT0027500	4.65	14.14	3.04	Not entried
miR-6802-5p	MIMAT0027504	21.82	69.40	3.18	Not entried
miR-6803-5p	MIMAT0027506	54.54	42.30	0.78	Not entried
miR-6805-5p	MIMAT0027510	79.35	50.59	0.64	Not entried
miR-6808-5p	MIMAT0027516	10.84	22.13	2.04	Not entried
miR-6809-5p	MIMAT0027518	4.89	17.84	3.65	Not entried
miR-6812-5p	MIMAT0027524	23.97	36.31	1.51	Not entried
miR-6816-5p	MIMAT0027532	21.94	16.13	0.73	Not entried
miR-6819-5p	MIMAT0027538	23.17	25.37	1.10	Not entried
miR-6820-5p	MIMAT0027540	21.43	17.29	0.81	Not entried
miR-6821-5p	MIMAT0027542	33.61	26.65	0.79	Not entried

Table S1. *Cont*

miRNA Name	miRBase Mature miRNA ID	Signal Intensity *		FC †	Entry of DIANA Database ‡
		Young Group	Old Group		
miR-6824-5p	MIMAT0027548	6.65	23.45	3.52	Not entried
miR-6828-5p	MIMAT0027556	42.69	14.26	0.33	Not entried
miR-6829-5p	MIMAT0027558	5.06	8.38	1.66	Not entried
miR-6780b-5p	MIMAT0027572	65.74	77.54	1.18	Not entried
miR-6840-3p	MIMAT0027583	6.85	9.82	1.43	Not entried
miR-6845-5p	MIMAT0027590	47.53	28.04	0.59	Not entried
miR-6849-5p	MIMAT0027598	116.66	141.13	1.21	Not entried
miR-6850-5p	MIMAT0027600	82.84	58.68	0.71	Not entried
miR-6858-5p	MIMAT0027616	13.18	16.47	1.25	Not entried
miR-6859-5p	MIMAT0027618	31.62	10.56	0.33	Not entried
miR-6769b-5p	MIMAT0027620	8.19	14.89	1.82	Not entried
miR-6861-5p	MIMAT0027623	24.75	18.70	0.76	Not entried
miR-6869-5p	MIMAT0027638	163.77	104.85	0.64	Not entried
miR-6873-5p	MIMAT0027646	6.14	4.26	0.69	Not entried
miR-6875-5p	MIMAT0027650	7.20	25.59	3.55	Not entried
miR-6877-5p	MIMAT0027654	17.15	16.82	0.98	Not entried
miR-6879-5p	MIMAT0027658	27.18	42.68	1.57	Not entried
miR-6885-5p	MIMAT0027670	19.22	30.80	1.60	Not entried
miR-6889-5p	MIMAT0027678	7.67	7.61	0.99	Not entried
miR-6891-5p	MIMAT0027682	5.26	21.45	4.08	Not entried
miR-6893-5p	MIMAT0027686	31.72	32.23	1.02	Not entried
miR-7106-5p	MIMAT0028109	7.46	34.93	4.68	Not entried
miR-7107-5p	MIMAT0028111	19.69	22.37	1.14	Not entried
miR-7108-5p	MIMAT0028113	75.85	18.29	0.24	Not entried
miR-7109-5p	MIMAT0028115	23.41	36.03	1.54	Not entried
miR-7111-5p	MIMAT0028119	12.02	25.92	2.16	Not entried
miR-7150	MIMAT0028211	6.62	20.65	3.12	Not entried
miR-7641	MIMAT0029782	13.13	26.83	2.04	Not entried
miR-7704	MIMAT0030019	254.84	264.86	1.04	Not entried
miR-4433b-3p	MIMAT0030414	5.69	11.31	1.99	Not entried
miR-7846-3p	MIMAT0030421	23.28	11.49	0.49	Not entried
miR-7847-3p	MIMAT0030422	8.38	17.47	2.08	Not entried
miR-8059	MIMAT0030986	5.54	18.68	3.37	Not entried
miR-8060	MIMAT0030987	13.41	20.93	1.56	Not entried
miR-8069	MIMAT0030996	197.54	82.31	0.42	Not entried
miR-8071	MIMAT0030998	16.01	10.84	0.68	Not entried
miR-8072	MIMAT0030999	350.39	213.48	0.61	Not entried
miR-8073	MIMAT0031000	5.91	11.51	1.95	Not entried
miR-128-2-5p	MIMAT0031095	21.53	45.64	2.12	Not entried

* Signal intensities were calculated by global normalization (ratio median = 1) method; † Fold change of miRNAs for old group using young group as reference; ‡ We used DIANA miRPath v.2.0. DIANA database were referring to miRBase 18. FC: fold change.

Table S2. NOD value of miRNAs and list of unique target genes.

miRNA Name	NOD *	Unique Target Genes
miR-124-3p	22	*LRP6*, *ROCK1*, *VANGL1*, *CCND2*, *ROCK2*, *FZD4*, *SMAD4*, *NFATC1*, *MAPK10*, *GNA13*, *GRIA2*, *RYR1*, *PTPN1*, *LIPE*, *RHOQ*, *GYS1*, *MAPK14*, *WASF1*, *MYH10*, *IQGAP1*, *DIAPH1*, *PGF*
miR-4739	20	*FGF6*, *ITGA8*, *FGF23*, *VAV3*, *IL2RG*, *TNFRSF11A*, *CNTFR*, *TNFSF13B*, *CXCL5*, *CCL11*, *IL22RA1*, *IL6R*, *ATP6AP1*, *AP4E1*, *GM2A*, *CTSO*, *ATP6V0B*, *AP1S1*, *AP1G1*, *GLB1*
miR-371a-5p	15	*PSEN1*, *LEF1*, *NAA38*, *C1D*, *DCP2*, *BTG3*, *PABPC5*, *CRNKL1*, *CDC40*, *SRSF2*, *SRSF7*, *SRSF6*, *AQR*, *SRSF3*, *WBP11*
miR-4530	13	*TAOK3*, *CACNG8*, *CRK*, *MST1*, *RASA1*, *JMJD7-PLA2G4B*, *NTRK1*, *MEF2C*, *FGFR1*, *PSEN2*, *SLC9A1*, *ATP1A4*, *ATP2A2*
miR-4728-5p	13	*RASA2*, *MAP2K7*, *MAP3K2*, *RRAS*, *PARVG*, *SHC1*, *ZYX*, *RHOA*, *ITGA11*, *PIK3R3*, *COL11A2*, *FLT4*, *COL5A3*
miR-4763-3p	13	*MAP3K11*, *MAP2K6*, *FGF18*, *JUND*, *MAPK8IP3*, *PPM1B*, *CACNA2D1*, *TGFBR2*, *PTK2B*, *PDE1B*, *PHKA1*, *SPHK2*, *LIFR*
miR-24-3p	12	*MYC*, *TCF7*, *IL1R1*, *TAB2*, *FASLG*, *TAOK1*, *FGF11*, *HSPA8*, *IL1A*, *SRF*, *RAP1B*, *CRH*
miR-4429	12	*KRAS*, *PRKCB*, *TSC1*, *PDK1*, *PRKAB2*, *AKT3*, *YWHAE*, *RAC1*, *ITGA1*, *PPP1R12A*, *PTEN*, *XIAP*
miR-204-3p	10	*CACNA1F*, *ERBB4*, *NMNAT1*, *ABCB9*, *GALNS*, *SUMF1*, *CD68*, *ARSG*, *CTSB*, *AP3S2*
miR-4459	10	*DVL3*, *NFATC4*, *MAPK9*, *FZD1*, *COL24A1*, *ITGB6*, *COL6A6*, *COL3A1*, *COL1A2*, *COL4A6*
miR-4516	8	*FZD7*, *TCF4*, *PPP2R5C*, *CACNG6*, *ITGA9*, *FGD3*, *PIP4K2B*, *ARHGEF4*
miR-4651	8	*FZD8*, *PRKCG*, *WNT7B*, *FGF17*, *PIK3R2*, *ARHGEF1*, *VAV1*, *LIMK1*
miR-762	8	*TCF7L1*, *PLCB3*, *NFATC2*, *FOSL1*, *PPARD*, *PPP3R2*, *PTPN5*, *RPS6KA4*
miR-1290	7	*GYS2*, *RIPK2*, *EGFR*, *PIP5K1B*, *ITGAV*, *SRC*, *CAPN2*
miR-1915-3p	7	*WNT2B*, *PPP2R5D*, *EP300*, *WNT3A*, *WNT9A*, *ADRA1B*, *VDAC3*
miR-3679-5p	7	*PRKAG1*, *PRKACB*, *PLCG1*, *ATP2B1*, *HTR5A*, *EDNRA*, *HTR2C*
miR-4667-5p	7	*RAF1*, *IRAK4*, *NGFR*, *EZR*, *ITGA5*, *ARHGEF6*, *ITGA7*
miR-920	7	*INHBB*, *IL2RB*, *EXOSC3*, *SKIV2L*, *PGAM4*, *PGAM1*, *PCK2*
miR-149-3p	6	*DUSP2*, *PFN1*, *SSH1*, *INSRR*, *MYH14*, *NCKAP1L*
miR-4478	6	*IL12RB1*, *IL13*, *CCR5*, *TNFRSF10B*, *CXCL9*, *CTF1*
miR-671-5p	6	*CXCL14*, *CSF2RB*, *RELT*, *IL17RB*, *KIT*, *IL17A*
miR-1224-5p	5	*PPP2R5A*, *PRICKLE2*, *ATP6V1H*, *AP1S2*, *IGF2R*
miR-3162-5p	5	*FGF12*, *DUSP22*, *GADD45A*, *PPM1A*, *RASGRP4*
miR-4484	5	*GNA11*, *CALM2*, *EIF4E*, *PAK7*, *ENAH*

Table S2. *Cont*

miRNA Name	NOD *	Unique Target Genes
miR-491-5p	5	*GNAS*, *LHCGR*, *ATP2B3*, *CACNA1D*, *HTR7*
miR-1246	4	*PLCB4*, *PRLR*, *EDA*, *ACVR1*
miR-30c-2-3p	4	*NAMPT*, *ATP1B2*, *SLC8A1*, *CACNB2*
miR-3175	4	*DAAM2*, *PABPC1L2B*, *PABPC1L2A*, *ENO2*
miR-3188	4	*AGT*, *ADH1B*, *GPI*, *CEL*
miR-3619-3p	4	*CCL4L2*, *CCL4L1*, *CCL4*, *FCGR2B*
miR-4726-5p	4	*CYSLTR2*, *ITPR2*, *ATP1B1*, *TPM4*
miR-197-5p	3	*ATP6V0D1*, *SCARB2*, *NDUFA2*
miR-4270	3	*TSC2*, *MAPK3*, *VCL*
miR-4646-5p	3	*WEE2*, *TFDP2*, *ORC5*
miR-4758-5p	3	*RPS17*, *RPS17L*, *RPS20*
miR-1254	2	*ADCY7*, *PHKG2*
miR-3197	2	*CTNNBIP1*, *ESPL1*
miR-3665	2	*NT5C*, *NNT*
miR-4497	2	*MCM5*, *RPS4X*
miR-4534	2	*CSF2RA*, *BMP7*
miR-4655-5p	2	*ARHGDIA*, *YWHAB*
miR-4665-5p	2	*MAP3K13*, *DUSP9*
miR-4674	2	*ADCY2*, *HTR2A*
miR-4689	2	*NUDT12*, *NT5C2*
miR-4734	2	*CD38*, *CUL1*
miR-1228-5p	1	*HIST1H4H*
miR-3131	1	*ABL1*
miR-3185	1	*PTGER3*
miR-3196	1	*ABCA2*
miR-345-3p	1	*DUSP6*
miR-3610	1	*TNFRSF10D*
miR-365a-5p	1	*SMAD2*
miR-3937	1	*C1QA*
miR-3940-5p	1	*SENP2*
miR-3960	1	*P2RX4*
miR-4281	1	*RPS6KB2*
miR-4486	1	*CACNA1A*
miR-4649-5p	1	*DDX39B*
miR-4723-5p	1	*PIP5K1C*
miR-4730	1	*TBL1X*
miR-4745-5p	1	*GRIN2A*
miR-4751	1	*G6PC*
miR-551b-5p	1	*CAMK2D*
miR-744-5p	1	*CALML5*

* Modified novel out degree (NOD) calculated by using 122 miRNAs commonly detected in the microarray analysis and entered in the DIANA database, and by using age-related pathways previously reported (Wang, 2012).
